# Effect of Larval Competition on Extrinsic Incubation Period and Vectorial Capacity of *Aedes albopictus* for Dengue Virus

**DOI:** 10.1371/journal.pone.0126703

**Published:** 2015-05-07

**Authors:** Jeffrey Bara, Zoi Rapti, Carla E. Cáceres, Ephantus J. Muturi

**Affiliations:** 1 Illinois Natural History Survey, University of Illinois, Champaign, Illinois, United States of America; 2 Department of Mathematics, University of Illinois, Urbana, Illinois, United States of America; 3 School of Integrative Biology, University of Illinois, Urbana, Illinois, United States of America; Instituto Nacional de Salud Pública, MEXICO

## Abstract

Despite the growing awareness that larval competition can influence adult mosquito life history traits including susceptibility to pathogens, the net effect of larval competition on human risk of exposure to mosquito-borne pathogens remains poorly understood. We examined how intraspecific larval competition affects dengue-2 virus (DENV-2) extrinsic incubation period and vectorial capacity of its natural vector *Aedes albopictus*. Adult *Ae*. *albopictus* from low and high-larval density conditions were orally challenged with DENV-2 and then assayed for virus infection and dissemination rates following a 6, 9, or 12-day incubation period using real-time quantitative reverse transcription PCR. We then modeled the effect of larval competition on vectorial capacity using parameter estimates obtained from peer-reviewed field and laboratory studies. Larval competition resulted in significantly longer development times, lower emergence rates, and smaller adults, but did not significantly affect the extrinsic incubation period of DENV-2 in *Ae*. *albopictus*. Our vectorial capacity models suggest that the effect of larval competition on adult mosquito longevity likely has a greater influence on vectorial capacity relative to any competition-induced changes in vector competence. Furthermore, we found that large increases in the viral dissemination rate may be necessary to compensate for small competition-induced reductions in daily survivorship. Our results indicate that mosquito populations that experience stress from larval competition are likely to have a reduced vectorial capacity, even when susceptibility to pathogens is enhanced.

## Introduction

Dengue fever is a rapidly emerging arthropod-borne virus (arbovirus) primarily transmitted to humans by two container-dwelling mosquito species, *Aedes aegypti* and *Ae*. *albopictus* [1], [2]. Annually, an estimated 100–390 million people become infected with one of the four closely related dengue virus serotypes (DENV 1–4) resulting in approximately 500,000 hospitalizations and 25,000 deaths [1, 3, 4]. With no immediate prospects for an effective vaccine or antiviral treatment, current dengue prevention efforts depend on vector control strategies which integrate the use of insecticides with natural enemies, elimination of breeding sites, and physical barriers such as house screening [5, 6]. Despite these efforts, reducing DENV incidence through mosquito control continues to be a significant challenge due in part to our limited understanding of the ecological factors that regulate DENV transmission and their relative importance in disease prevention and management.

For a mosquito to transmit DENV, it must acquire a blood meal from an infected host, support viral replication and dissemination to the salivary glands (vector competence), survive the extrinsic incubation period (EIP, the duration between acquisition of an infectious agent and the potential to transmit that agent), and take a subsequent blood meal from a susceptible host. The length of the EIP has a significant effect on the temporal dynamics of DENV transmission and may be affected by a variety of environmental factors including the fitness of the infecting genotype, viral titer of the infectious blood meal, and temperature [7–10]. Under natural conditions, the EIP of many mosquito-borne pathogens is long relative to mosquito lifespan, and less than 10% of female mosquitoes are estimated to survive the EIP to become potential vectors [11]. The EIP of DENV is estimated to be between 8–12 days [4, 9, 12] while the mean lifespan of *Ae*. *albopictus* females is estimated to be between 3–11 days [13–16]. Therefore, slight changes in either EIP or mosquito longevity can have significant consequences on the ability of *Ae*. *albopictus* to transmit DENV [11, 17].

Vectorial capacity describes the capacity of a vector population to transmit a pathogen and takes into account tripartite interactions between host, virus, and vector [17–21]. An accurate estimate of vectorial capacity is necessary to evaluate vector control strategies and predict the risk of pathogen transmission [19, 21]. Vectorial capacity (*C*) is expressed as:

$$C=\frac{ma^{2}bp^{n}}{-ln(p)}$$

Where ***m*** is the ratio of mosquitoes to humans, ***a*** is the human biting rate, ***b*** is vector competence, ***p*** is the daily survival rate of the vector, and ***n*** is the EIP.

The parameters of vectorial capacity vary spatially and temporally depending on biotic and abiotic environmental fluctuations, and an individual environmental factor can affect multiple vectorial capacity parameters in complex or antagonistic ways [22]. For example, elevated temperatures may enhance vector competence (***b***) and decrease EIP *(* ***n***) while concomitantly reducing adult longevity (***p***) [10,23,24]. Competition for limited resources may also reduce adult mosquito lifespan (***p***) while enhancing vector competence (***b***) [25, 26]. Understanding how environmental factors affect the relationship between the components of vectorial capacity, especially ***p*** and ***n***, is therefore essential for accurate estimation of disease transmission risk.

A growing body of knowledge has shown that adult mosquito life history traits are largely influenced by environmental conditions experienced during larval development [23–33]. These studies have stimulated interest in understanding the contribution of the larval environment to the risk of mosquito-borne pathogen transmission [28–31, 34]. However, our understanding of how these interactions affect the vectorial capacity of mosquito populations is limited, due in part to the complex ways in which the larval environment affects the ability of adults to transmit pathogens.

Larval competition is an ubiquitous ecological stressor that has the potential to affect the vectorial capacity of mosquito populations [25, 26, 28, 34]. *Ae*. *albopictus* larvae develop in a variety of artificial water holding containers (e.g. water tanks, discarded tires, trash cans, flower vases) [14, 35] that derive most of their energy through decomposition of allochthonous detrital inputs, primarily leaf litter [15, 34, 36]. Larvae in these habitats often attain very high densities and populations are usually regulated by intra- and interspecific resource competition [28, 34, 37]. When larval competition is severe enough to affect larval survivorship, it can reduce adult emergence rates and in turn vector density (***m***). Further, surviving mosquitoes that reach adulthood may have altered behavior, physiology, or life history traits that could affect their potential to vector pathogens. For example, stress from larval competition often results in the production of smaller adults, which is correlated with reduced longevity (***p***) [27, 38–41], altered biting rates (***a***) [40, 42, 43], and higher vector competence (***b***) [25, 28–30].

Larval competition can also influence viral virulence because it often yields adult mosquitoes with a shorter lifespan, thereby reducing future transmission opportunities. Consequently, competition could select for viral strains with rapid replication, shorter EIPs (***n***) and higher virulence [44]. Alternatively, if larval competition results in enhanced viral susceptibility and diminished physiological barriers to dissemination and transmission, it could reduce viral virulence by reducing the force of selection.

In this study, we evaluated the effect of intraspecific larval competition on the EIP of DENV-2 in *Ae*. *albopictus*. We then used the cumulative vectorial capacity equation (cVC) developed by Christofferson and Mores [20] to assess the net effect of larval competition on vectorial capacity of *Ae*. *albopictus* for DENV. We tested the hypotheses that: 1) larval competition would enhance *Ae*. *albopictus* susceptibility to DENV-2 and shorten the EIP for this virus, and 2) the effects of larval competition on adult mosquito longevity will outweigh its effects on vector competence, and ultimately have an overall negative effect on vectorial capacity.

## Materials and Methods

### Competition study

We combined a laboratory competition and infection experiment with mathematical models to investigate the effect of intraspecific larval competition on EIP and vectorial capacity of *Ae*. *albopictus* for DENV-2. The competition experiment was conducted using F_15_ and F_16_ generations of *Ae*. *albopictus* from field collections in Florida. First-instar larvae (< 24 hr old) were added to 1.6 liters of filtered oak infusion held in 5-liter plastic containers at an initial larval density of 150 or 300 per container. The containers were maintained at 25°C. Each container was supplemented with 0.2 g of larval food (1:1 albumin: yeast) on day 1 and 0.05 g on days 7 and 10 of the experiment. Each container was examined daily until all larvae had either pupated or died. Pupae from each replicate were removed daily and placed into plastic vials with water until eclosion. Newly emerged adults (both males and females) from each container (replicate) were housed in paperboard cages (11 cm high x 9.5 cm diameter) by date of emergence. There were 4 biological replicates for each larval density-EIP combination for a total of 24 containers.

### Vector competence study

*Aedes albopictus* epithelial cells (C6/36) (ATCC # CRL-1660) were maintained at 35°C in Leibovitz L-15 media (Invitrogen, Carlsbad, CA), supplemented with 10% fetal bovine serum (FBS) (Atlanta Biological, Norcross GA) and 10 μg/ml of penicillin and streptomycin (Invitrogen, Carlsbad, CA). We used DENV-2 strain 16803 (Southeast Asian genotype) in this study. This strain was isolated from a patient in Thailand in 1974 and was inoculated into *Toxorhynchites amboinensis* once, passaged three times in primary African green monkey kidney (PGMK) cells, twice in C6/36 cells and four times in Vero cells before we obtained this strain from Walter Reed Army Institute of Research. Confluent monolayers of C6/36 cells in 25-cm^2^ flasks were inoculated with DENV-2 at a multiplicity of infection of 0.1 and incubated at 35°C for 90 minutes with occasional rocking to allow for viral attachment and entry. Six milliliters of L-15 media was then added to each flask for a final volume of 6.3 ml/flask and incubated for 6 days. Freshly harvested C6/36 cell monolayers and media were used to prepare the infectious blood meals consisting of a mixture of citrated bovine blood (BB*100, HemoStat Laboratories, Dixon, CA) and media in a 3:2 ratio for a final DENV-2 titer of log 10^6.32^–10^6.49^ focus forming units (ffu) /ml.

Four to 7-day-old female *Ae*. *albopicus* that had been sugar-starved for 48 hours were provided 40 min access to the infectious blood meal via the Hemotek membrane feeding system (Lancashire, UK). Blood-fed females were transferred into paperboard cages and maintained on 10% sucrose at 28°C and 70% relative humidity for an incubation period of 6, 9, or 12 days. Following the incubation period, individual mosquitoes were killed and their legs and wings detached from their bodies. The wings were used for body size determination while the bodies and legs were preserved separately at -80°C and later assayed for DENV-2.

#### Quantification of DENV in infectious blood meal

Vero cells (ATCC # CCL-81) were propagated in L-15 medium (Invitrogen), supplemented with 10% FBS and 10 μg/ml of penicillin and streptomycin (Invitrogen) and maintained at 37°C and 5% CO_2_. Ten-fold serial dilutions of DENV-2 in L-15 media were inoculated in triplicate on confluent Vero cell monolayers in 24-well plates and incubated at 37°C for 90 minutes with periodic rocking to facilitate virus adsorption. Subsequently, wells were overlaid with 1 ml of 0.8% methylcellulose (Sigma-Aldrich, St. Louis, MO) diluted in warm L-15 media supplemented with 5% FBS, and 10 μg/ml of penicillin and streptomycin (Invitrogen), then incubated for 5 days at 37°C and virus plaques were visualized by immunoperoxidase staining as described by Durbin et al.[45].

#### DENV quantification in mosquitoes by real-time PCR

Bodies and legs of individual mosquitoes were separately homogenized in 1 ml of media prior to RNA extraction. Total RNA was extracted using Qiamp virus Biorobot 9604 kit according to manufacturer’s protocol (Qiagen, Valencia, CA). A one-step Taqman probe qRT-PCR was used to determine the infection status of body and leg samples as well as the body titer of infected mosquitoes. qRT-PCR was conducted in 20 μL reactions containing 10 μL of one-step Sensifast RT-PCR master mix (Bioline, Tauton, MA), 0.2 μL RNAse inhibitor, 0.8 μL of each 10 μM forward and reverse primer stock, 0.4 μL of 10 μM Taqman probe, 2.8 μL nuclease-free water (Integrated DNA Technologies, Coralville IA) and 5 μL template RNA. Primers and probe used for this study targeted the nonstructural protein 1 portion of DENV genome; forward primer (5’-CATGGCCCTKGTGGC-3’), reverse primer (5’-CCCCATCTYTTCAGTATCCCT -3’), and probe (5’ FAM-TCCTTCGTTTCCTAACAATCC-BHQ1-3’). qRT-PCR reactions were conducted in a 7300 real time PCR system (Applied Biosystems, Foster City, CA). Thermocycling conditions were: 48°C for 30 min, 95°C for 2 min followed by 45 cycles of 95°C for 15 s, 60°C for 1 min, and 72°C for 1 min. To determine DENV titer within mosquito bodies, total RNA was standardized to 40 ng/ml and qRT-PCR was conducted as described above. Threshold cycle (Ct) values were compared to a DENV-2 standard curve with Ct values generated from DENV-2 with a known titer (ffu/ml) in cell culture. *Ae*. *albopictus* with DENV-2 RNA in their bodies and legs were considered to have a disseminated infection, while those with an infected body and uninfected legs represent a non-disseminated infection. The body infection rate was calculated as the number of females with a non-disseminated infection divided by the number of mosquitoes exposed. The disseminated infection rate was calculated as the number of mosquitoes with DENV-2 RNA in their legs divided by the number of mosquitoes exposed.

### Vectorial capacity models

We used the cumulative vectorial capacity equation developed by Christofferson and Mores [17] to investigate the effect of larval competition on vectorial capacity. The cumulative vectorial capacity equation calculates effective vector competence (EVC), which is the rate of pathogen dissemination over time, instead of a single discrete value using the equation:

$$b_{i}(N)=\beta_{li}N+\beta_{0i}$$

Where b_i_(N) is the function for larval density i;β_1_ is the determined change in ***b*** per unit change in N; N is day post exposure; and β_0_ is the y-intercept. This line represents the rate of change in dissemination rate over time. Effective vector competence (*φ*), is defined as:

$$EVC\;=\;\varphi =\int_{a}^{z}p^{N}(\beta_{li}N+\beta_{0i})dN$$

The interval over which this line is constructed has a lower limit of time *a* and an upper limit of time *z*. The values of *a* and *z* in our study are 6 and 12 days, respectively. The EIP of DENV is believed to be between 8–12 days [4, 9, 11, 12], therefore our three time points measure early, middle, and late viral dissemination, respectively. Effective vector competence is then inserted into the cumulative vectorial capacity (cVC) defined as:

$$cVC=\frac{ma^{2}\varphi }{-ln(p)}$$

To address our second hypothesis, we generated three cVC models, based on parameter estimates derived from peer reviewed laboratory and field studies (Table 1). Small adult body size in mosquitoes is often correlated with shorter lifespan [39–41]. In our vectorial capacity models, we assumed that small mosquitoes from the high-density treatment would have a 0–6% decrease in daily survivorship based on values observed in field and laboratory studies (Table 2). Overall, there was a lack of peer-reviewed studies on the human biting rate (***a***) and mosquito density (***m***) for *Ae*. *albopictus*. Therefore we based our estimates of ***m*** and ***a*** from field studies on *Ae*. *aegypti*. For both parameters ***m*** and ***a***, there was a range of values reported in the literature and we based our parameter estimates on the approximate median of these values (Table 1). The vectorial capacity models assume a naïve end-host population with no significant vertical transmission [20]. In our first model we used our disseminated infection rate data to assess the effect of larval competition on cVC assuming equal daily survivorship (0.85) between the high and low density treatments, or a 5% higher daily survivorship (0.90) for the low density treatment, holding human-biting rate (***a*** = 2) and mosquito density constant (***m*** = 1.5). Second, we modeled the effect of larval competition on cVC using our disseminated infection rate data across a range of human-biting rates, which varied from 0–3, while holding mosquito density constant (***m*** = 1.5) and assuming equal survivorship (0.90 or 0.85), or a 1% (0.85 vs 0.84) difference in survivorship between the low and high density treatments, respectively. Lastly, we estimated the change in the viral dissemination rate that is necessary to compensate for a 3% or 6% decrease in the daily survivorship rate assuming ***a*** = 2 and ***m*** = 1.5.

**Table 1:** Vectorial Capacity model parameters.

Parameter	Value	References
**Probability of Daily Survival (p)**	0.85–0.90	[10–13, 46–49]
**Man Biting Rate (a)**	0–3	[50–52]
**Mosquito Density (m)**	1.5	[53, 54]
**Extrinsic Incubation Period (n)**	6–12 days	[4, 9, 12]
**Vector competence** ^a^ **(b)**	-	-

^a^ obtained from experimental data

**Table 2:** Estimated reduction in daily survivorship rate in small mosquitoes.

Species	Environmental stressor	Reduction in female daily survivorship rate	Study type	Reference
***Culex tarsalis***	intraspecific competition	0–3%^$^	laboratory	[53]
***Aedes albopictus***	intraspecific competition	2–4%*	laboratory	[41]
***Aedes aegypti***	diet modification	3–4%	field	[39]
***Aedes albopictus***	intraspecific competition	0–1%^#^	laboratory	[38]
***Aedes aegypti***	intraspecific competition	1–2%^#^	laboratory	[38]

^$^ based on larval density per container

* derived from mean LT_50_ and LT_90_ values from water + blood treatment

^#^ estimated from mean survivorship of low density and high density treatments at 85% humidity

### Statistical analysis

Statistical analyses were conducted using SAS 9.3 (SAS Corporation, Cary, NC). Data were checked for normality (Kolmogorov-Smirnov test) and homogeneity of variances (examination of residuals) and multivariate analysis of variance (MANOVA) was used to determine the effect of larval density on emergence rate, time to emergence, and female wing length. Standardized canonical coefficients were used to describe the relative contribution of larval density to the significant multivariate effect. Separate analysis of variance (ANOVAs) were used to determine the effect of larval density, incubation period, and their 2-way interaction on infection rate and viral titer respectively. A separate ANOVA was used to determine the effect of larval density on cVC assuming equal survivorship (0.85) for the high and low density treatments, or assuming a 5% higher daily survivorship rate (0.90) for the low density treatment. Separate logistic regressions were conducted by larval density, to test the relationship between female body size and body and disseminated infection rate. The probability of DENV infection (infected = 1, uninfected = 0) and infection status (disseminated infection = 1, non-disseminated infection = 0) was tested against the continuous variable wing length. For all analyses, significant main effects and interactions were followed by pairwise contrasts of least-square means using the Tukey-Kramer adjustment for multiple comparisons.

## Results

### Effect of larval density on adult life history traits

**Fig 1:**
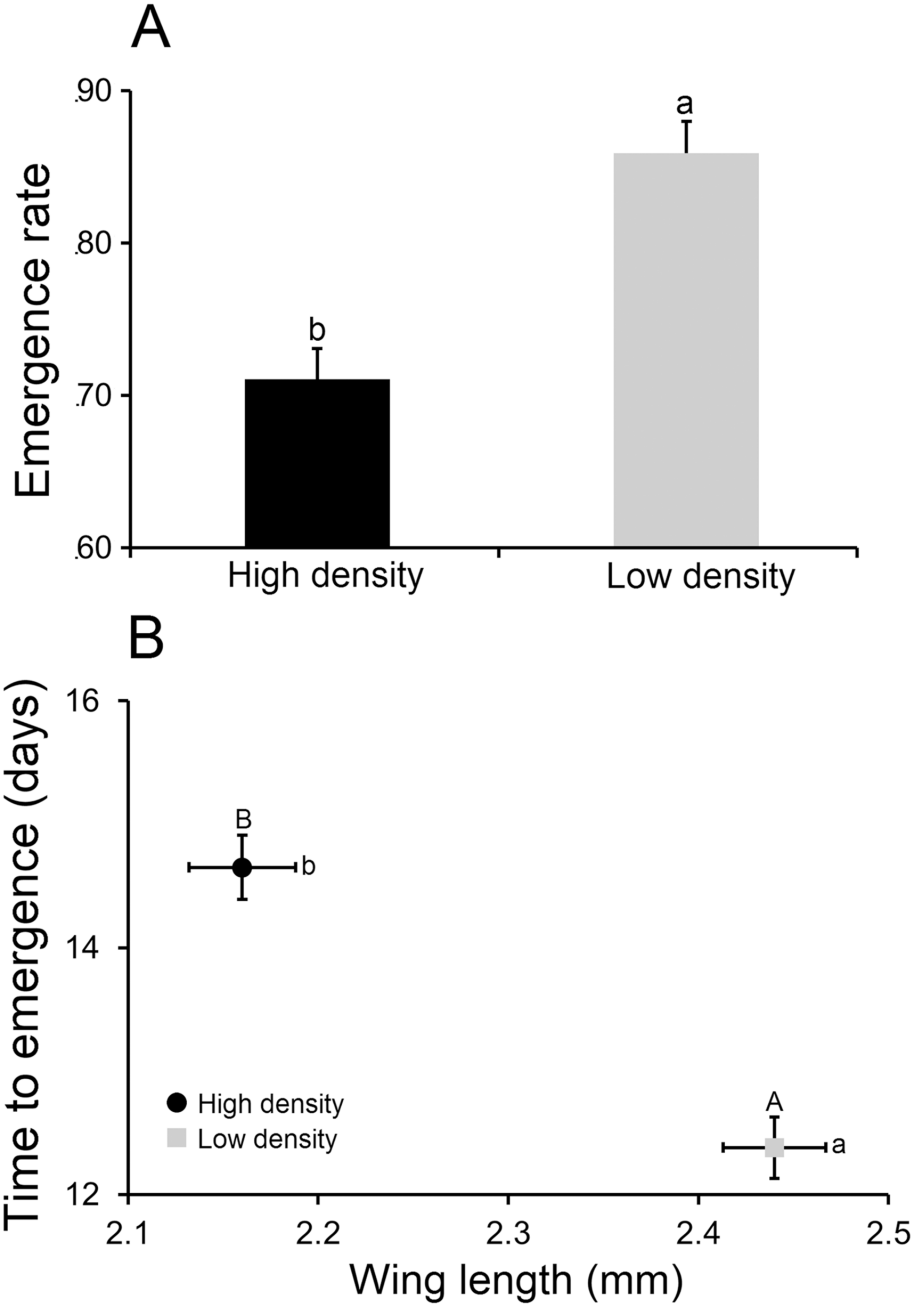
Effect of larval competition on *Aedes albopictus* life history traits. Panel A displays the effect of larval density on emergence rate. Panel B presents a bivariate plot of female time to emergence and female wing length. Error bars represent the standard errors of the mean. Tests of significance were corrected for multiple comparisons using the Tukey-Kramer adjustment. Bivariate means followed by different lower—and uppercase letters indicate significant differences in wing length and time to emergence respectively.

MANOVA revealed a significant effect of larval density on emergence rate, female time to emergence and wing length (S1 Table). Wing length contributed most to variation followed by emergence rate (Table 3). Emergence rates were significantly higher in the low-density treatment relative to the high-density treatment (ANOVA; F = 23.99; df = 1,19; *P* < 0.001) (Fig 1A). In addition, females from low-density treatment were significantly larger (ANOVA F = 34.37; df = 1,19; *P* < 0.001), and had a shorter time to emergence (ANOVA F = 31.17; df = 1,19; *P*<0.001) relative to females from the high-density treatment (Fig 1B).

**Table 3:** Multivariate ANOVA for the effect of larval density on emergence rate, female time to emergence, and female body size.

				Standardized canonical coefficients (SCC)		
Variable	df	Pillai's trace	*P*	Emergence rate	Time to emergence	Wing length
Larval Density	3,17	0.8605	<0.0001	1.229	-0.332	1.545

### *Ae*. *albopictus* susceptibility to DENV

**Fig 2:**
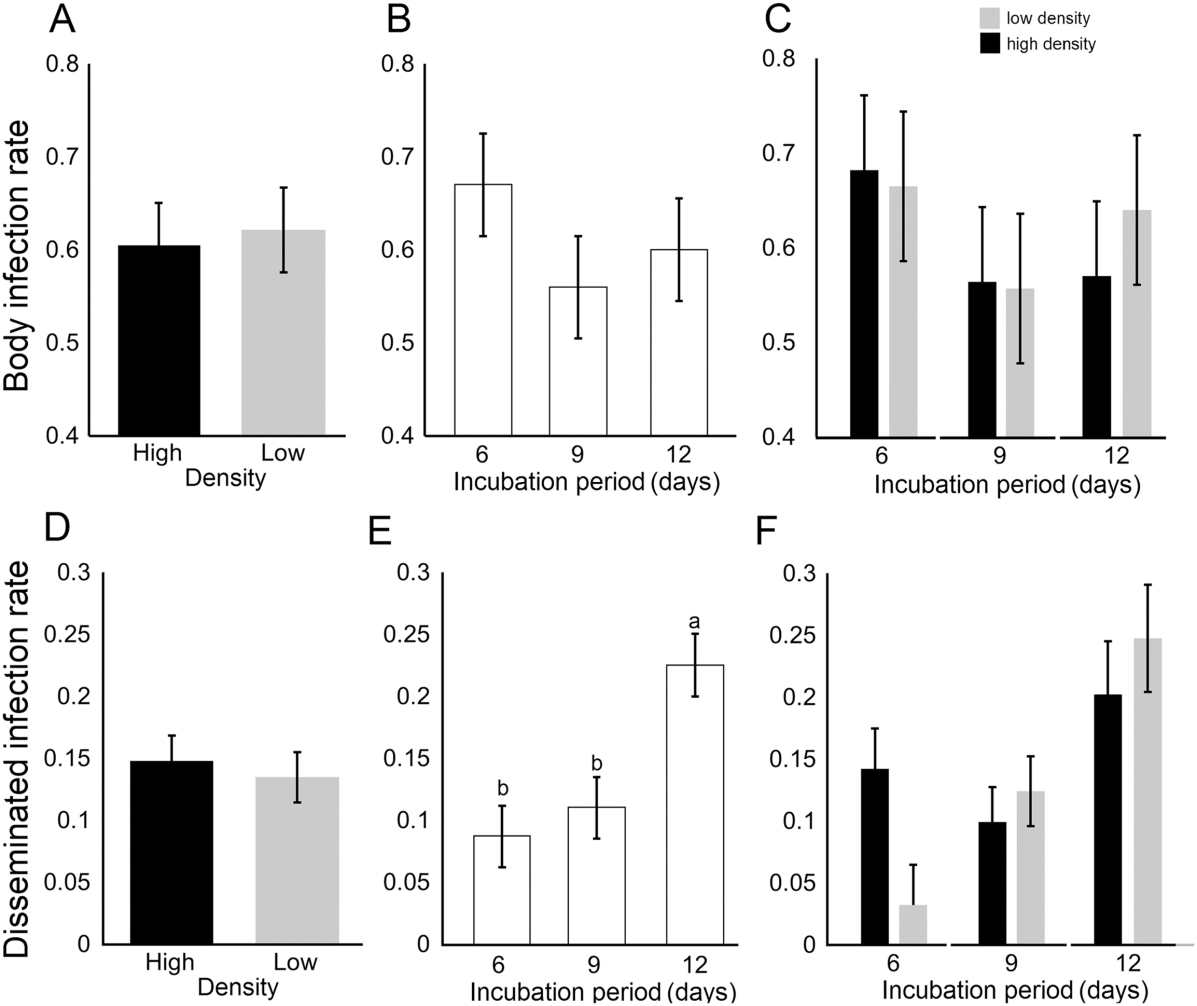
*Ae*. *albopictus* body and disseminated leg infection rates. Panel A-C shows the body infection rate by larval density, incubation period, and larval density by incubation period respectively. Panel D-F shows the disseminated infection rate by larval density, incubation period and larval density by incubation period. Bars above and below the means represent the standard errors of the mean. For each panel, estimates with a different letter are significantly different at *P* = 0.05, after controlling for multiple comparisons using the Tukey-Kramer adjustment.

**Fig 3:**
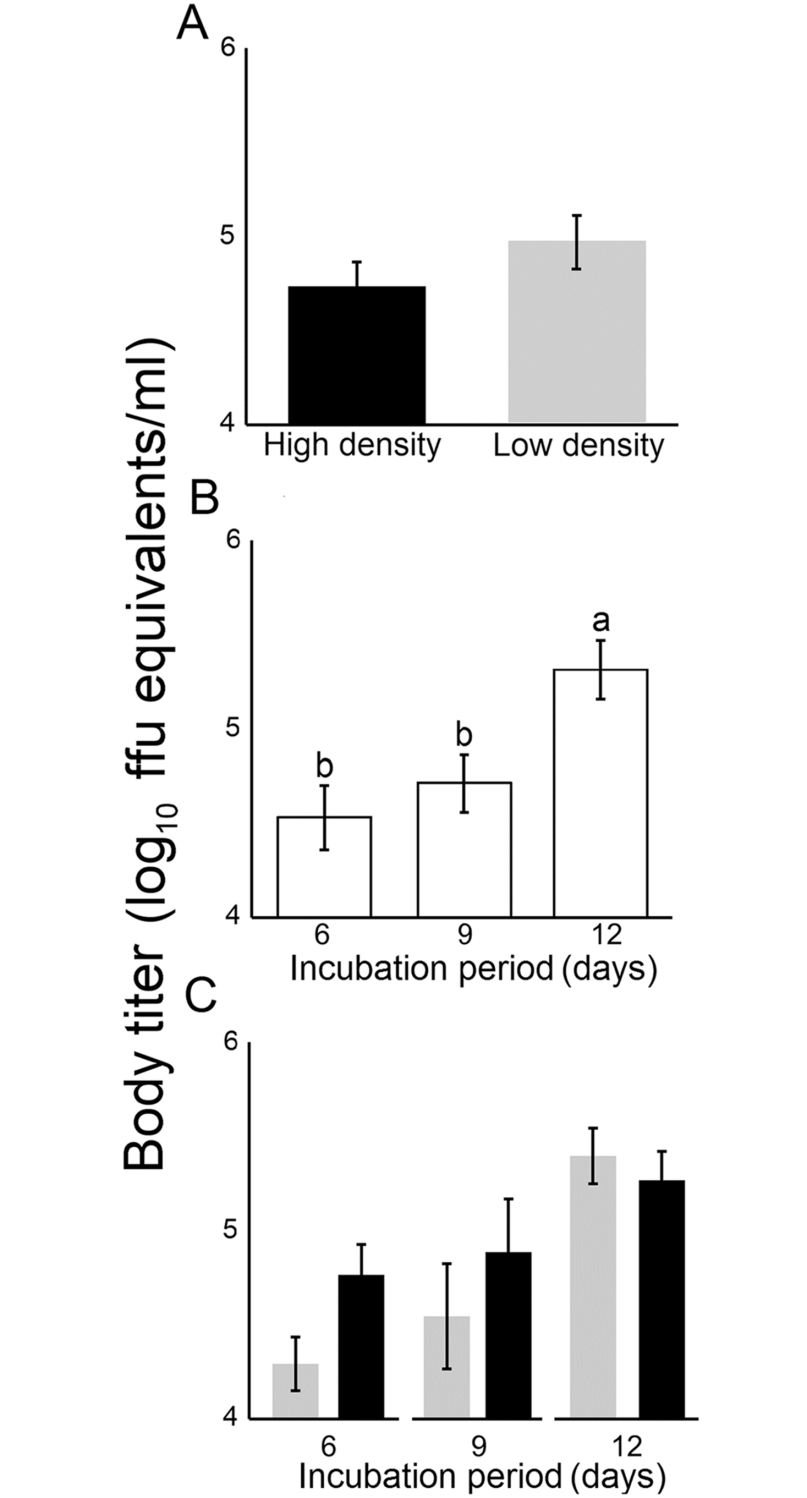
Effect of larval density (A), incubation period (B) and larval density by incubation period (C) on DENV titer in infected mosquito bodies. Bars above and below the means represent the standard errors of the mean. For each panel, estimates with a different letter are significantly different at *P* = 0.05, after controlling for multiple comparisons using the Tukey-Kramer adjustment.

A total of 496 adult *Ae*. *albopictus* females were assayed for DENV-2 infection and dissemination. The proportion of *Ae*. *albopictus* with a body infection was not significantly affected by larval density, incubation period or their interaction (Table 4; Fig 2A–2C). The proportion of *Ae*. *albopictus* with a disseminated infection was significantly affected by incubation period (IP) but not larval density (Fig 2D and 2E). DENV-2 dissemination rates were significantly higher at the 12-day IP compared to either the 6- (*P* = 0.002) or 9-day IP (*P* = 0.01; Fig 2E). The larval density by incubation period interaction was not significant but depicted a trend of higher DENV dissemination rates at higher compared to lower larval density at the 6-day IP but not at 9 or 12-day IP (Table 4, Fig 2F). DENV-2 titer in infected *Ae*. *albopictus* females was not significantly influenced by larval density (Fig 3A) or the incubation period by larval density interaction (Table 4). However, the length of the incubation period had a significant effect on viral titer (Fig 3B). DENV-2 titer in mosquitoes was significantly higher at 12-day IP relative to either 6-day (*P* = 0.001) or 9-day IP dpe (*P* = 0.01).

**Table 4:** Univariate ANOVA for the effect of larval density, incubation period, and larval density by incubation period on midgut infection rate, disseminated infection rate and viral titer.

**Body** infection rate						
Model term	Source	numDF	denDF	F-value	P-value	Significance
Main effect	Larval density (LD)	1	18	0.06	0.81	N.S.
Main effect	EIP	2	18	1.03	0.37	N.S.
Interaction	EIP*LD	2	6	0.18	0.832	N.S.
Disseminated infection rate						
**Model term**	**Source**	**numDF**	**denDF**	**F-value**	**P-value**	**Significance**
Main effect	Larval density (LD)	1	18	0.21	0.65	N.S.
Main effect	EIP	2	18	8.64	**0.0023**	**
Interaction	EIP*LD	2	6	2.84	0.08	N.S.
Viral titer (body)						
**Model term**	**Source**	**numDF**	**denDF**	**F-value**	**P-value**	**Significance**
Main effect	Larval density (LD)	1	18	2.32	0.145	N.S.
Main effect	EIP	2	18	10.2	**0.001**	***
Interaction	EIP*LD	2	6	1.21	0.322	N.S.

N.S. p>0.05;

* p<0.05;

** 0.001<p<0.01;

*** p<0.001

#### Relationship between body size and vector competence

At both high and low-larval density treatments, the likelihood of a body infection was not influenced by female body size. However, for the high-density treatment, the probability of a disseminated infection was inversely related to body size (Table 5).

**Table 5:** Logistic regression assessing the relationship between DENV infection and dissemination status and female body size by larval density.

Infection status	Source	Density	df	Logit (SE)	t-value	P-value
**Body infection**		High				
	intercept		11	0.950 (1.906)	0.50	0.627
	size		266	-0.115 (0.862)	-0.13	0.8933
**Disseminated infection**		High				
	intercept		11	4.163 (2.472)	1.68	0.1202
	size		174	-2.371 (1.131)	-2.10	0.0375
**Body infection**		Low				
	intercept		11	2.337 (1.764)	1.32	0.2121
	size		126	-0.648 (0.712)	-0.91	0.3641
**Disseminated infection**		Low				
	intercept		11	1.466 (2.362)	0.62	0.547
	size		126	-1.090 (0.965)	-1.13	0.26

### Vectorial Capacity models

**Fig 4:**
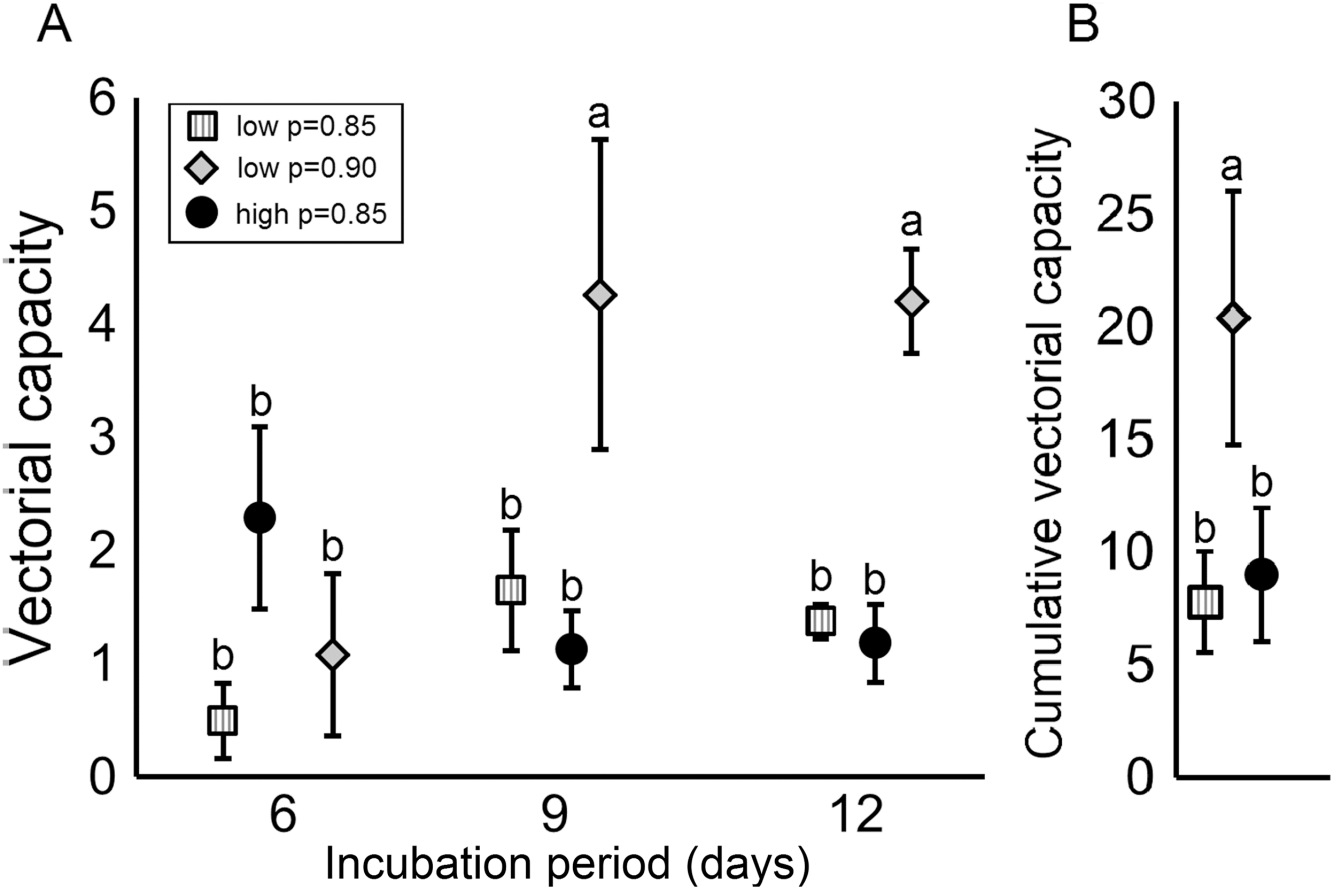
Effect of larval competition on vectorial capacity. Panel A displays the vectorial capacity values for high and low density treatments at the 6, 9, and 12-day incubation periods. Panel B displays the cumulative vectorial capacity for high and low-density treatments. Tests of significance were corrected for multiple comparisons using the Tukey-Kramer adjustment. Different letters indicate significant differences in means.

**Fig 5:**
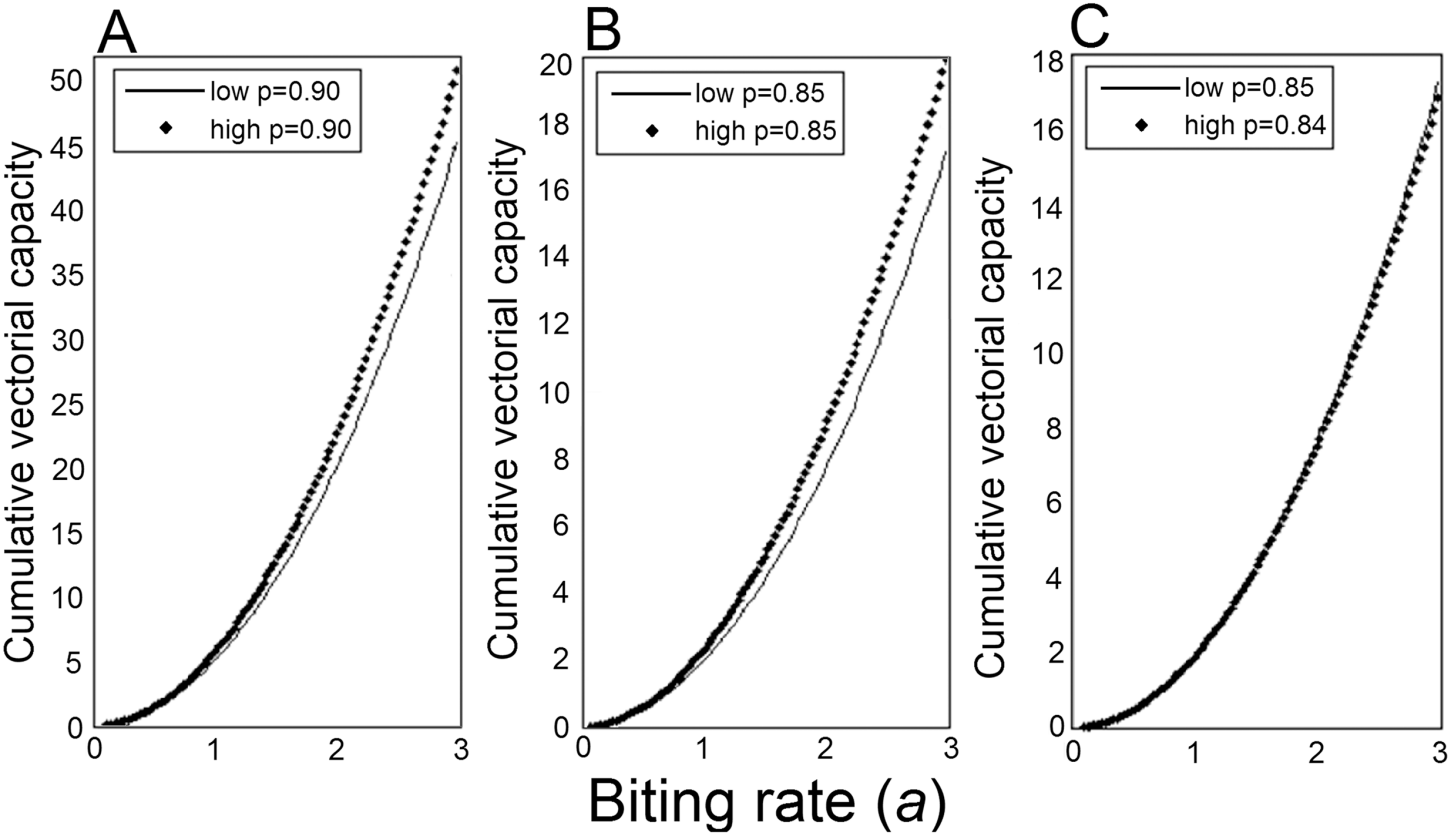
Cumulative vectorial capacity of high and low density treatments across a range of human biting rates.

**Fig 6:**
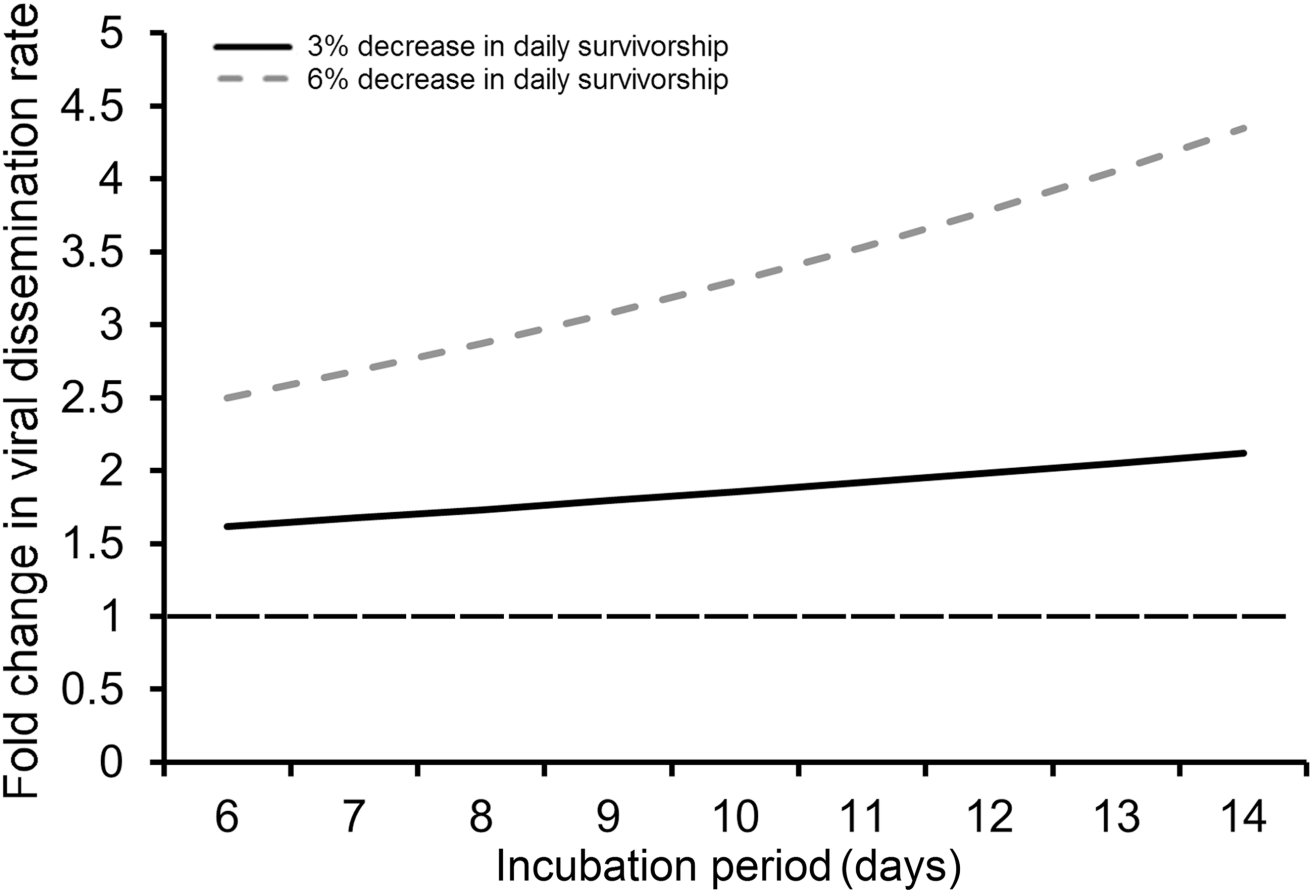
Change in viral dissemination rate necessary to compensate for decrease in daily survivorship rate. The figure above displays the change in viral dissemination rate necessary to compensate for a reduction in the daily survivorship rate by the specified amount from an estimated 90% survival rate.

Assuming an equal survivorship of 0.85 for adult females from both high and low-density treatments, there was a marginally significant effect of larval competition on vectorial capacity at the 6-day IP (F = 5.65, df = 1,6; *P* = 0.055) but not at the 9 (F = 0.94, df = 1,6; *P* = 0.37) or 12-day IP (F = 0.36, df = 1,6; *P* = 0.56) or cVC (F = 0.20, df = 1,7; *P* = 0.66) (Fig 4A and 4B). However, when we assumed that larger adult mosquitoes from low-density treatment had a 0.90 daily survival rate compared to 0.85 for smaller mosquitoes from high larval density, there was a significant effect of larval competition on vectorial capacity at the 9-day IP (F = 6.58, df = 1,6; *P* = 0.042), 12-day IP (F = 37.11, df = 1,6; *P* = 0.0009) as well as on cVC (F = 21.07, df = 1,7; *P* = 0.0013). Mosquitoes from low-density treatment had significantly higher vectorial capacity at the 9 and 12-day IP as well as higher cVC (Fig 4A and 4B). As shown in our second model, a 5% decrease in daily survivorship has a significant effect on cVC reducing it by a magnitude of at least 2.5 fold from 50 to 20 for low density treatment and 45 to 17 for high density treatment (Fig 5A and 5B). Secondly, differences in cVC become more prominent as the biting rate increases. At biting rates below 1 the cVC of the high and low-density populations are equivalent (Fig 5A and 5B). Overall, the high-density treatment had a higher cVC when equal survivorships of 0.85 and 0.90 were assumed (Fig 5A and 5B). When a 1% difference in survivorship was assumed for mosquitoes from low (0.85) and high (0.84) density treatments an equal cVC between the two populations was observed (Fig 5C). In our third model, we calculated the fold change in viral dissemination necessary to compensate for a 3% or 6% decrease in survivorship, assuming a 90% daily survivorship. To compensate for a 3% decrease in daily survivorship, the viral dissemination rate would need to increase by 1.6 to 2.1 fold across a 6–14 day incubation period, while a 2.5 to 4.3 fold increase is necessary to compensate for a 6% decrease in survivorship across a 6–14 day incubation period (Fig 6).

## Discussion

In this study we tested the hypotheses that: 1) larval competition would enhance *Ae*. *albopictus* susceptibility to DENV-2 and shorten its EIP, and 2) the effects of larval competition on longevity will outweigh its effects on vector competence, and ultimately have an overall negative effect on vectorial capacity. Our results weakly supported our first hypothesis and strongly supported our second hypothesis providing evidence that larval competition may have a significant effect on the vectorial capacity of mosquito populations.

Increasing larval density two-fold resulted in a significantly longer time to emergence, lower emergence rates, and smaller females. These findings are consistent with previous observations that intraspecific larval competition can negatively impact adult mosquito fitness both at individual [41, 55, 56], and population level [29, 50, 57]. How larval competition impacts vectorial capacity is primarily driven by its effects on daily survivorship, the most important determinant of a mosquito’s ability to transmit pathogens [19, 58]. We found that slight reductions in longevity that were not statistically significant may still have important biological consequences. Additionally, the fitness cost of DENV infection may be greater in small females, which have lower nutrient reserves relative to large females [27], further contributing to differences in longevity between large and small mosquitoes.

Larval competition had no significant effect on the susceptibility of *Ae*. *albopictus* to DENV-2. The effect of larval competition on the susceptibility of mosquitoes to arboviruses remains unclear as there is evidence to suggest that larval competition decreases [59, 60] has no effect [61] or increases mosquito susceptibility to arboviruses [28–30]. We hypothesized that smaller mosquitoes from the high-density treatment would have enhanced susceptibility to DENV based on previous observations that small mosquitoes may have reduced immune function [56] and reduced physiological barriers to viral dissemination [62]. However, our results did not support our hypothesis. This could be attributed in some measure to the differences in experimental design as all previous studies utilized a single time point to assess vector competence. Though our results did not support our hypothesis, they do suggest that larval competition may alter mosquito-virus interactions as we found a significant correlation between body size and viral dissemination among mosquitoes in the high-density treatment.

Our findings partially supported our hypothesis that larval competition would shorten the DENV-2 EIP in *Ae*. *albopictus*. We observed a trend of earlier viral dissemination in mosquitoes from the high density treatment at the 6-day IP that resulted in a 4.5 fold increase in vectorial capacity relative to the low-density treatment. Although this result narrowly failed statistical significance (*P* = 0.055) under the conditions of our experiment, it could have significant biological implications in nature. First, viral dissemination at earlier incubation periods greatly increases the number of females that may survive to become infectious. Second, early viral dissemination in mosquitoes can alter the temporal dynamics of pathogen transmission by reducing the latent period between vertebrate hosts. Third, by reducing mosquito longevity, larval competition would be expected to select for viral strains with the shortest EIP in mosquitoes [44]. This could have important implications for human health as DENV isolates with short EIPs in mosquitoes are associated with more severe clinical symptoms in humans [7].

One of the difficulties in evaluating the impact of altered vector competence on vectorial capacity is the degree to which other life history traits are affected. Reductions in adult body size, presumably due to a stressful larval environment are associated with lower biting rates, higher viral dissemination rates, and lower daily survivorship [30, 39–41]. These trade-offs are rarely evaluated properly when mosquitoes are tested for their susceptibility to an arbovirus, and it is often assumed that enhanced vector competence will increase the vectorial capacity of mosquito populations. However, longevity and vector competence are not equivalent terms in the vectorial capacity equation; longevity is part of an exponential term, while vector competence is a linear term [17, 19]. To develop a better understanding of the relationship between longevity and vector competence, we estimated the change in the viral dissemination rate necessary to compensate for a 3% or 6% decrease in daily survivorship. We found that after a 14 day EIP, a 2-fold increase in the viral dissemination rate is necessary to compensate for a 3% decrease in daily survivorship. This increase in DENV susceptibility greatly exceeds the level of enhanced susceptibility reported in experimental studies [28–30]. We did not incorporate associated changes in the biting rate or mosquito density in this model in part due to the lack of peer-reviewed studies on *Ae*. *albopictus*. Low larval densities yield higher adult emergence rates and larger *Ae*. *albopictus*, which may have a higher host attack rate relative to small mosquitoes [40]. Therefore by holding these two parameters constant, our model underestimates the negative effect of competition on vectorial capacity. Thus for most mosquito-borne pathogens, environmental factors that reduce female longevity are likely to decrease vectorial capacity, even when susceptibility is enhanced [40, 63]. These results are in agreement with recent studies by Araujo et al. [64], Moller-Jacobs et al. [65], Juliano et al. [66], and Breaux et al. [63] in indicating that stressful conditions within the larval environment yield adult mosquitoes with a reduced capacity to vector pathogens.

We also found that increases in viral dissemination rate over time often may not necessarily translate to increased vectorial capacity. Due to cumulative effect of adult mortality, increases in viral dissemination rate that occur at later incubation periods (> 9 days) become more unlikely to affect vectorial capacity. For example, although the viral dissemination rate of the high and low density treatments increased approximately two-fold from the 9 to 12-day IP, the vectorial capacity for both treatments actually decreased over this time period (S1 Fig). Similarly, Christofferson and Mores [20], found that DENV-2 strain with the highest disseminated infection rate in *Ae*. *aegypti* did not have the highest cVC, since the increase in viral dissemination rate for this strain primarily occurred after a 9-day IP. Alternatively, increases in the viral dissemination rate at early incubation periods (< 6 days) are more likely to affect vectorial capacity. This suggests that the biological relevance of vector competence studies is highly dependent on the use of relevant incubation periods for the vector-virus system.

Vector control remains the primary tool for reducing the incidence of vector-borne diseases.

In the face of global climate change, and expanding geographic ranges of mosquito species and the diseases they vector [6, 10, 67–69], improving quantitative assessment tools and mathematical models are necessary to accurately predict disease risks and improve vector control programs [21]. Determining the direction and magnitude of larval competition on vectorial capacity has important consequences for vector control programs. If competitive interactions have a negative effect on vectorial capacity, control measures (e.g. larvicides) that result in less than 100% mortality will release surviving larvae from competition, resulting in the production of larger adults with a higher vectorial capacity [70, 71]. Additionally, determining which artificial container habitats support *Ae*. *aegypti* and *Ae*. *albopictus* populations with the greatest capacity for DENV transmission can substantially improve the efficiency and cost effectiveness of mosquito control programs [6].

Some aspects of our study may influence how our results are translated to other systems. First, we used DENV RNA in mosquito legs as a proxy for the presence of DENV in the salivary glands, which marks the end of the EIP [9]. The presence of viral RNA in mosquito legs is highly correlated with viral dissemination into the head and salivary glands, but could potentially over estimate the number of infectious mosquitoes [72]. Second, given the lack of peer reviewed studies on the daily biting rates (a) and mosquito density (m) for *Ae*. *albopictus*, we used studies based on *Ae*. *aegypti* to estimate both parameters. While this approach provides insights into the potential consequences for larval competition on DENV vectorial capacity, further studies are necessary to generate the vectorial capacity parameters for *Ae*. *albopoctus*. Thirdly, as vector competence is influenced by genotype*genotype interactions, conclusions drawn from one vector-virus combination may not be representative of other vector-virus systems under a variety of environmental conditions [73]. Lastly, this study was conducted using a laboratory strain of *Ae*. *albopictus* which may differ in susceptibility to DENV relative to natural populations [74]. However, our aim was to test for the effect of larval competition on DENV vectorial capacity and there is no evidence that laboratory colonization alters the effect of larval competition on vector susceptibility to DENV. For this reason, we believe our results provide an accurate indication of how larval competition may influence the vectorial capacity of this important vector. Additional studies using field collected populations of *Ae*. *albopictus* are needed to validate these findings.

In summary, our data suggest that larval competition has a weak effect on the EIP of DENV but can significantly alter the vectorial capacity of *Ae*. *albopictus*. In addition, our vectorial capacity models supported the hypothesis that stress-mediated decreases in the daily survivorship rate are likely to reduce the vectorial capacity of a mosquito population even if vector competence is enhanced. The effect of larval competition on adult fitness is context-dependent and may vary depending species interactions, detritus type or quantity, presence of anthropogenic chemicals (e.g. insecticides, pesticides) and temperature [24, 28, 75]. Therefore, additional studies investigating the effect of larval competition on arbovirus EIP and vector longevity under different environmental conditions are needed to understand how this ubiquitous ecological stressor affects transmission of mosquito-borne diseases.

## Supporting Information

S1 Fig The relationship between viral dissemination rate and vectorial capacity across the 6–12 day incubation period. (TIF)

S1 Table Mean life history traits, infection rates, and cumulative vectorial capacity values for the high and low-density treatments. (TIF)

## References

[1] Kyle JL, Harris E (2008) Global spread and persistence of dengue. Annu Rev Microbiol 62: 71–92. 10.1146/annurev.micro.62.081307.163005 18429680

[2] Vasilakis N, Deardorff ER, Kenney JL, Rossi SL, Hanley KA, Weaver SC (2009) Mosquitoes put the brake on arbovirus evolution: experimental evolution reveals slower mutation accumulation in mosquito than vertebrate cells. PLoS Pathog 5: e1000467 10.1371/journal.ppat.1000467 19503824 PMC2685980

[3] Chen R, Vasilakis N (2011) Dengue—Quo tu et quo vadis? Viruses 3: 1562–1608. 10.3390/v3091562 21994796 PMC3187692

[4] Guzman MG, Halstead SB, Artsob H, Buchy P, Farrar J, Gubler DJ, et al (2010) Dengue: a continuing global threat. Nat Rev Microbiol 8: S7–16. 10.1038/nrmicro2460 21079655 PMC4333201

[5] World Health Organization and Special Programme for Research and Training in Tropical Diseases (2009) Dengue: Guideline for diagnosis, treatment, prevention and control. France: World Health Organization

[6] Wong J, Morrison AC, Stoddard ST, Astete H, Chu YY, Baseer I, et al (2012) Linking oviposition site choice to offspring fitness in *Aedes aegypti*: Consequences for targeted larval control of dengue vectors. PLoS Neglected Tropical Diseases 6: e1632 10.1371/journal.pntd.0001632 22563512 PMC3341338

[7] Anderson JR, Rico-Hesse R (2006) *Aedes aegypti* vectorial capacity is determined by the infecting genotype of dengue virus. Am J Trop Med Hyg 75: 886–892. 17123982 PMC1993907

[8] Pesko K, Westbrook CJ, Mores CN, Lounibos LP, Reiskind MH (2009) Effects of infectious virus dose and bloodmeal delivery method on susceptibility of *Aedes aegypti* and *Aedes albopictus* to Chikungunya virus. J Med Entomol 46: 395–399. 19351094 10.1603/033.046.0228 PMC2716074

[9] Chan M, Johansson MA (2012) The incubation periods of dengue viruses. PLoS ONE 7: e50972 10.1371/journal.pone.0050972 23226436 PMC3511440

[10] Liu-Helmersson J, Stenlund H, Wilder-Smith A, Rocklo J (2014) Vectorial capacity of *Aedes aegypti*: Effects of temperature and implications for global dengue epidemic potential. PLoS ONE 9: e89783 10.1371/journal.pone.0089783 24603439 PMC3946027

[11] Joy TK, Jeffrey Gutierrez EH, Ernst K, Walker KR, Carriere Y, Torabi M, et al (2012) Aging field collected *Aedes aegypti* to determine their capacity for dengue transmission in the Southwestern United States. PLoS ONE 7: e46946 10.1371/journal.pone.0046946 23077536 PMC3470585

[12] Watts DM, Burke DS, Harrison BA, Whitmire RE, Nisalak A (1987) Effect of temperature on the vector efficiency of *Aedes aegypti* for dengue 2 virus. Am J Trop Med Hyg 36: 143–152. 3812879 10.4269/ajtmh.1987.36.143

[13] Mori A (1979) Effects of larval density and nutrition on some attributes of immature and adult *Aedes albopictus* . Trop Med 21: 85–103.

[14] Delatte H, Toty C, Boyer S, Bouetard A, Bastien F, Fontenille D (2013) Evidence of habitat structuring *Aedes albopictus* populations in Réunion Island. PLoS Neglected Tropical Diseases 7: e2111 10.1371/journal.pntd.0002111 23556012 PMC3605158

[15] Hawley WA (1988) The biology of *Aedes albopictus* . J Am Mosq Control Assoc S1: 1–40. 3068349

[16] Niebylski ML, Craig GB (1994) Dispersal and survival of *Aedes albopictus* at a scrap tire yard in Missouri. J Am Mosq Control Assoc 10: 339–343. 7807074

[17] Dye C (1986) Vectorial capacity: must we measure all its components? Parasitology Today 2: 203–209. 15462840 10.1016/0169-4758(86)90082-7

[18] Garrett-Jones C, Shidrawi GR (1969) Malaria vectorial capacity of a population of *Anopheles gambiae*: an exercise in epidemiological entomology. Bull World Health Organ 40: 531–545. 5306719 PMC2556109

[19] Kramer LD, Ebel GD (2003) Dynamics of flavivirus infection in mosquitoes. Adv Vir Res 60: 187–232. 10.1016/s0065-3527(03)60006-0 14689695

[20] Christofferson RC, Mores CN (2011) Estimating the magnitude and direction of altered arbovirus transmission due to viral phenotype. PLoS ONE 6:e16298 10.1371/journal.pone.0016298 21298018 PMC3029343

[21] Bellan SE (2010) The importance of age dependent mortality and the extrinsic incubation period in models of mosquito-borne disease transmission and control. PLoS ONE 5: e10165 10.1371/journal.pone.0010165 20405010 PMC2854142

[22] Lefèvre T, Vantaux A, Dabiŕe KR, Mouline K, Cohuet A (2013) Non-genetic determinants of mosquito competence for malaria parasites. PLoS Pathog 9: e1003365 10.1371/journal.ppat.1003365 23818841 PMC3688545

[23] Tabachnick WJ (2013) Nature, nurture and evolution of intra-species variation in mosquito arbovirus transmission competence. Int J Environ Res Pub Heal 10: 250–277. 10.3390/ijerph10010249 PMC3564141 23343982

[24] Alto BW, Bettinardi D (2013) Temperature and dengue virus infection in mosquitoes: independent effects on the immature and adult stages. Am J Trop Med 88: 497–505. 10.4269/ajtmh.12-0421 23382163 PMC3592531

[25] Alto BW, Lounibos LP, Higgs S, Juliano SA (2005) Larval competition differentially affects arbovirus infection in *Aedes* mosquitoes. Ecology 86: 3279–3288. 19096729 10.1890/05-0209 PMC2605070

[26] Alto BW, Bettinardi DJ, Ortiz S (2015) Interspecific larval competition differentially impacts adult survival in dengue vectors. J Med Entomol 52: 163–170. 26336301 10.1093/jme/tju062

[27] Briegel H, Timmermann SE (2001) *Aedes albopictus* (Diptera:Culicidae): physiological aspects of development and reproduction. J Med Entomol 38: 566–571. 11476337 10.1603/0022-2585-38.4.566

[28] Alto BW, Lounibis LP (2013) Vector competence for arboviruses in relation to the larval environment of mosquitoes In: Takken W, Koenraadt CJM, editors. Ecology and control of vector-borne diseases. Netherlands: Wageningen Academic Publishers pp 81–101.

[29] Alto BW, Lounibos LP, Mores CN, Reiskind MH (2008) Larval competition alters susceptibility of adult *Aedes* mosquitoes to dengue infection. Pro Roy Soc B 275: 463–71. 10.1098/rspb.2007.1497 PMC2289994 18077250

[30] Alto BW, Reiskind MH, Lounibos LP (2008) Size alters susceptibility of vectors to dengue virus infection and dissemination. Am J Trop Med 79: 688–695. 18981505 PMC2630770

[31] Muturi EJ, Blackshear M, Montgomery A (2012) Temperature and density-dependent effects of larval environment on *Aedes aegypti* competence for an alphavirus. J Vect Ecol 37: 154–161. 10.1111/j.1948-7134.2012.00212.x 22548549

[32] Westbrook CJ, Reiskind MH, Pesko KN, Greene KE, Lounibos LP (2010) Larval environmental temperature and the susceptibility of *Aedes albopictus* Skuse (Diptera: Culicidae) to Chikungunya virus. Vector-borne Zoonotic Dis 10: 241–247. 10.1089/vbz.2009.0035 19725768 PMC2883477

[33] Bevins SN (2007) Timing of resource input and larval competition between invasive and native container-inhabiting mosquitoes (Diptera: Culicidae). J Vect Ecol 32: 252–262. 10.3376/1081-1710(2007)32[252:torial]2.0.co;2 18260515

[34] Juliano SA (2009) Species interactions among larval mosquitoes: context dependence across habitat gradients. Annu Rev Entomol 54: 37–56 10.1146/annurev.ento.54.110807.090611 19067629 PMC2664081

[35] Paupy C, Delatte H, Bagny L, Corbel V, Fontenille D (2009) *Aedes albopictus*, an arbovirus vector: From the darkness to the light. Microbe Infect 11: 1177–1185. 10.1016/j.micinf.2009.05.005 19450706

[36] Yee DA, Allgood D, Kneitel JM, Kuehn KA (2012) Constitutive differences between natural and artificial container mosquito habitats: vector communities, resources, microorganisms, and habitat parameters. J Med Entomol 49: 482–491. 22679854 10.1603/me11227

[37] Washburn J (1995) Regulatory factors affecting larval mosquito populations in container and pool habitats: implications for biological control. J Am Mosq Control Assoc 11: 279–283. 7595462

[38] Reiskind MH, Lounibos LP (2009) Effects of intraspecific larval competition on adult longevity in the mosquitoes *Aedes aegypti* and *Aedes albopictus* . Med Vet Entomol 23: 62–68. 10.1111/j.1365-2915.2008.00782.x 19239615 PMC2651082

[39] Maciel-de-Freitas R, Codeco CT, Lourenco-de-Oliveira R (2007) Body size-associated survival and dispersal rates of *Aedes aegypti* in Rio de Janeiro. Med Vet Entomol 21: 284–292. 17897370 10.1111/j.1365-2915.2007.00694.x

[40] Nasci RS (1986) The size of emerging and host-seeking *Aedes aegypti* and the relation of size to blood-feeding success in the field. J Am Mosq Control Assoc 2: 61–62. 3507471

[41] Xue RD, Barnard DR, Muller GC (2010) Effects of body size and nutritional regimen on survival in adult *Aedes albopictus* (Diptera: Culicidae). J Med Entomol 47: 778–782. 20939370 10.1603/me09222

[42] Xue RD, Barnard DR, Schreck CE (1995) Effect of body size, parity, and age of *Aedes albopictus* on human host attack rates and the repellency of deet. J Am MosqControl Assoc 11: 50–53. 7616190

[43] Nasci RS (1991) Influence of larval and adult nutrition on biting persistence in *Aedes aegypti* (Diptera: Culicidae). J Med Entomol 28: 522–526. 1941913 10.1093/jmedent/28.4.522

[44] Nidelet T, Koella JC, Kaltz O (2009) Effects of shortened host life span on the evolution of parasite life history and virulence in a microbial host-parasite system. BMC Evol Biol 9: 65 10.1186/1471-2148-9-65 19320981 PMC2666652

[45] Durbin AP, Karron RA, Sun W, Vaughn DW, Reynolds MJ, Perreault JR, et al (2001) Attenuation and immunogenicity in humans of a live dengue virus type-4 vaccine candidate with a 30 nucleotide deletion in its 3′-untranslated region. Am J Trop Med Hyg 65: 405–413. 11716091 10.4269/ajtmh.2001.65.405

[46] Lambrecht FL (1971) Notes on the ecology of Seychelles mosquitoes. Bull Entomol Res 60: 513–532.

[47] Makiya K (1974) Population dynamics of mosquitoes in Nagoya district. B. Larval and imaginal populations of *Aedes albopictus* (Skuse) in a cemetery of Nagoya City. Jap J Sanit Zool 25: 41–49.

[48] Gould DJ, Mount GA, Scanlon JE, Ford HR, Sullivan MF (1970) Ecology and control of dengue vectors on an island in the gulf of Thailand. J Med Entomol 7:499–508. 5530313 10.1093/jmedent/7.4.499

[49] Toma T, Sakamoto S, Miyagi I (1982) The seasonal appearance of Aedes albopictus in Okinawajima, the Ryukyu archipelago, Japan. Mosq News 42:179–183.

[50] Focks DA, Brenner RJ, Hayes J, Daniels E (2000) Transmission thresholds for dengue in terms of *Aedes aegypti* per person with discussion of their utility in source reduction efforts. Am J Trop Med Hyg 62: 11–18. 10761719

[51] Yasuno M, Tonn RJ (1970) A study of biting habits of *Aedes aegypti* in Bangkok, Thailand. Bull World Health Organ 43: 319–325. 5312528 PMC2427649

[52] Scott TW, Amerasinghe PH, Morrison AC, Lorenz LH, Clark GG, Strickman D, et al (2000) Longitudinal studies of *Aedes aegypti* (Diptera: Culicidae) in Thailand and Puerto Rico: Blood feeding frequency. J Med Entomol 37: 89–101. 15218911 10.1603/0022-2585-37.1.89

[53] Reisen WK, Milby MM, Bock ME (1984) The effects of immature stress on selected events in the life history of *Culex tarsalis* . Mosq News 44: 385–395.

[54] Jeffery JAL, Yen NT, Nam VS, Nghia LT, Hoffmann AA, Kay BH, et al (2009) Characterizing the *Aedes aegypti* population in a vietnamese village in preparation for a wolbachia-based mosquito control strategy to eliminate dengue. PLoS Negl Trop Dis 3: e0000552 10.1371/journal.pntd.0000552 19956588 PMC2780318

[55] Murrell EG, Juliano SA (2008) Detritus type alters the outcome of interspecific competition between *Aedes aegypti* and *Aedes albopictus* . J Med Entomol 45: 375–383. 18533429 10.1603/0022-2585(2008)45[375:dtatoo]2.0.co;2 PMC2583230

[56] Telang A, Qayum AA, Parker A, Sacchetta BR, Byrnes GR (2012) Larval nutritional stress affects vector immune traits in adult yellow fever mosquito *Aedes aegypti* (*Stegomyia aegypti*). Med Vet Entomol 26: 271–281. 10.1111/j.1365-2915.2011.00993.x 22112201

[57] Lord CC (1998) Density dependence in larval *Aedes albopictus* (Diptera: Culicidae). J Med Entomol 35: 825–829. 9775616 10.1093/jmedent/35.5.825

[58] Styer LM, Carey JR, Wang JL, Scott TW (2007) Mosquitoes do senesce: departure from the paradigm of constant morality. Am J Trop Med Hyg 76: 111–117. 17255238 PMC2408870

[59] Bevins SN (2008) Invasive mosquitoes, larval competition, and indirect effects on the vector competence of native mosquito species (Diptera: Culicidae). Biol Invasion 10: 1109–1117.

[60] Sumanochitrapon W, Strickman D, Sithiprasasna R, Kittayapong P, Innis BL (1998) Effect of size and geographic origin of *Aedes aegypti* on oral infection with dengue-2 virus. Am J Trop Med Hyg 58:283–286. 9546404 10.4269/ajtmh.1998.58.283

[61] Dodson BL, Kramer LD, Rasqon JL (2011) Larval nutritional stress does not affect vector competence for West Nile virus (WNV) in *Culex tarsalis* . Vector-Borne Zoonotic Diseases 11: 1493–1497. 10.1089/vbz.2011.0662 21867417 PMC3216062

[62] Grimstad PR, Walker ED (1991) *Aedes triseriatus* (Diptera: Culicidae) and La Crosse virus IV nutritional deprivation of larvae affects the adult barriers to infection and transmission. J Med Entomol 28: 378–386. 1875364 10.1093/jmedent/28.3.378

[63] Breaux JA, Schumacher MK, Juliano SA (2014) What does not kill them males them stronger: Larval environment and infectious dose alter mosquito potential to transmit filarial worms. P Roy Soc B 281: 20140459. 10.1098/rspb.2014.0459 PMC4046410 24827444

[64] Araujo M, Gil LH, e-Silva A (2012) Larval food quantity affects development time, survival and adult biological traits that influence the vectorial capacity of *Anopheles darlingi* under laboratory conditions. Malaria J 11: 261 10.1186/1475-2875-11-261 22856645 PMC3469369

[65] Moller-Jacobs LL, Murdock CC, Thomas MB (2014) Capacity of mosquitoes to transmit malaria depends on larval environment. Parasites and Vectors 7:593 25496502 10.1186/s13071-014-0593-4 PMC4273441

[66] Juliano SA, Ribeiro GS, Maciel-de-Freitas R, Castro MG, Lourenco-de-Oliveira R, Lounibos LP (2014) She’s a femme fatale: low-density larval development produces good disease vectors. Mem Inst Oswaldo Cruz 109:1070–1077. 10.1590/0074-02760140455 25591112 PMC4325623

[67] Esteva L, Vargas C (2000) Influence of vertical and mechanical transmission on the dynamics of dengue disease. Math Biosci 167: 51–64. 10942786 10.1016/s0025-5564(00)00024-9

[68] Morin CW, Comrie AC, Ernst K (2013) Climate and dengue transmission: evidence and implications. Environ Health Perspect 121:1264–1272. 10.1289/ehp.1306556 24058050 PMC3855512

[69] Morrison AC, Zielinski-Gutierrez E, Scott TW, Rosenberg R (2008) Defining challenges and proposing solutions for control of the virus vector *Aedes aegypti* . PLoS Medicine 5(3): e68 10.1371/journal.pmed.0050068 18351798 PMC2267811

[70] Agudelo-Silva F, Spielman A (1984) Paradoxical effects of simulated larviciding on production of adult mosquitoes. Am J Trop Med Hyg 33:1267–126. 6507734 10.4269/ajtmh.1984.33.1267

[71] Arrivillaga J, Barrera R (2004) Food as a limiting factor for *Aedes aegypti* in water-storage containers. J Vect Ecol 29: 11–20. 15266737

[72] Turell MJ, O’Guinn ML, Dohm DJ, Jones JW (2001) Vector competence of North American mosquitoes (Diptera: Culicidae) for West Nile virus. J Med Entomol 38: 130–134. 11296813 10.1603/0022-2585-38.2.130

[73] Lambrechts L, Chevillon C, Albright RG, Thaisomboonsuk B, Richardson JH, Jarman RG, et al (2009) Genetic specificity and potential for local adaptation between dengue viruses and mosquito vectors. BMC Evol Biol 9:160 10.1186/1471-2148-9-160 19589156 PMC2714696

[74] Lambrechts L, Scott TW, Gubler DJ (2010) Consequences of the expanding global distribution of *Aedes albopictus* for dengue virus transmission. PLoS Negl Trop Dis 4: e646 10.1371/journal.pntd.0000646 20520794 PMC2876112

[75] Muturi EJ, Alto BW (2011) Larval environmental temperature and insecticide exposure alter *Aedes aegypti* competence for arboviruses. Vector-Borne and Zoonotic Diseases, 11: 1157–11 10.1089/vbz.2010.0209 21453010

