# Supplementary figures and images for: Effect of Larval Competition on Extrinsic Incubation Period and Vectorial Capacity of Aedes albopictus for Dengue Virus
Source: PLoS One. 2015 May 7;10(5):e0126703. doi: 10.1371/journal.pone.0126703 (PMC4423876; doi:10.1371/journal.pone.0126703)

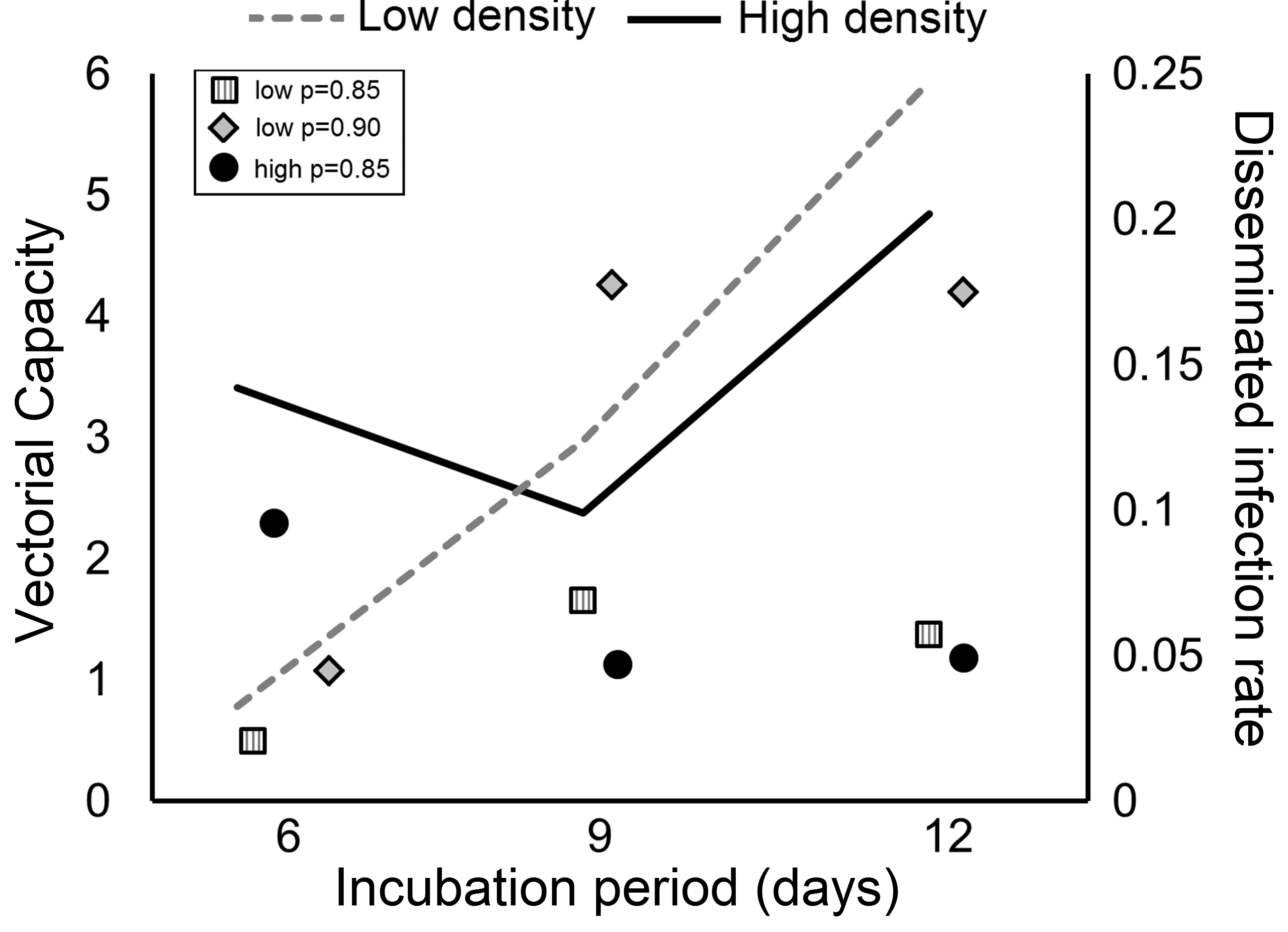

Supplement: S1 Fig — (TIF) [file pone.0126703.s001.tif]

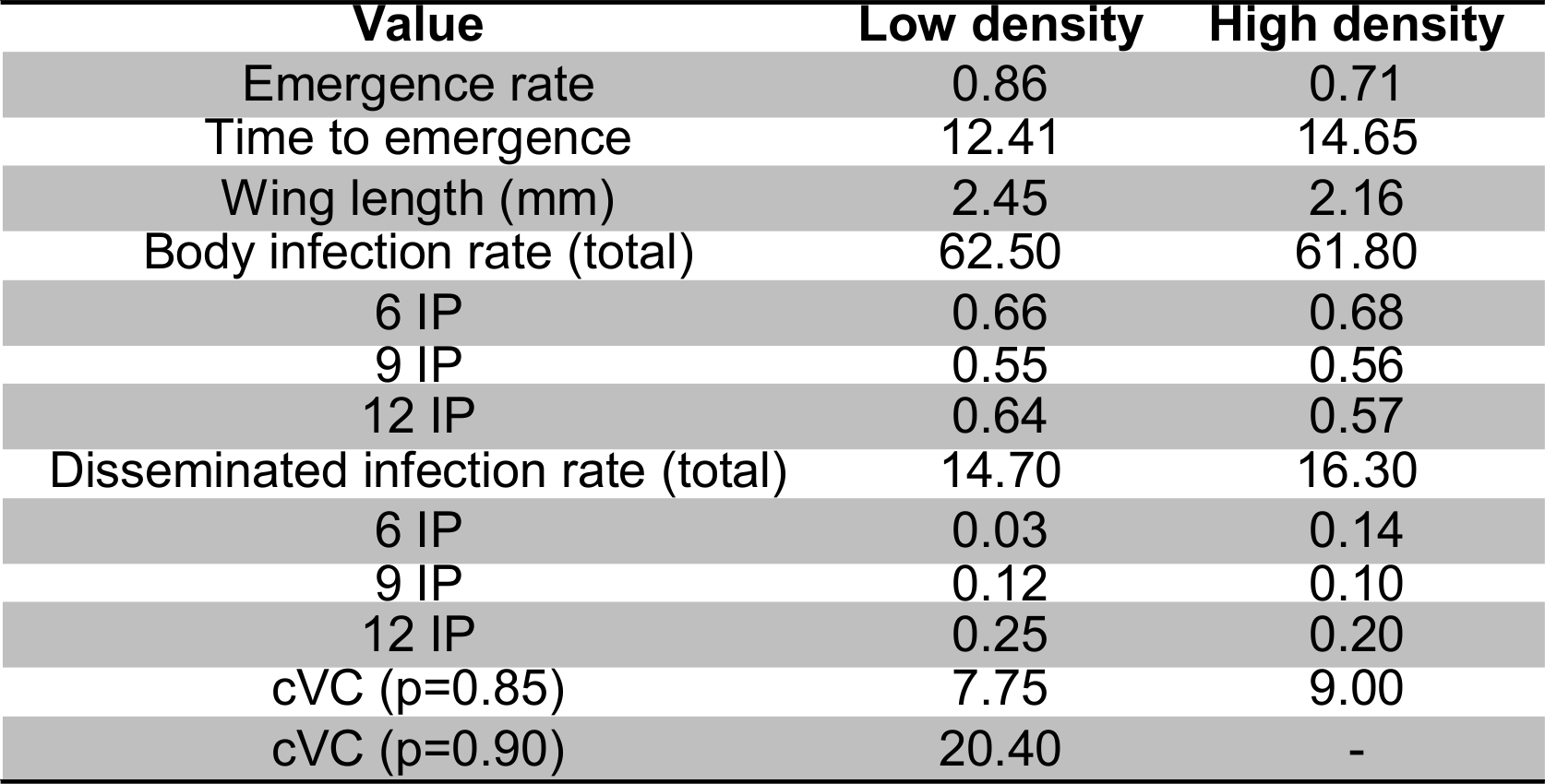

Supplement: S1 Table — (TIF) [file pone.0126703.s002.tif]
